# Nutrient-dependent regulation of β-cell proinsulin content

**DOI:** 10.1016/j.jbc.2023.104836

**Published:** 2023-05-19

**Authors:** Xiaoxi Xu, Anoop Arunagiri, Maroof Alam, Leena Haataja, Charles R. Evans, Ivy Zhao, Roberto Castro-Gutierrez, Holger A. Russ, Caroline Demangel, Ling Qi, Billy Tsai, Ming Liu, Peter Arvan

**Affiliations:** 1 Division of Metabolism, Endocrinology & Diabetes, University of Michigan Medical Center, Ann Arbor, Michigan, USA; 2 Department of Endocrinology and Metabolism, Tianjin Medical University General Hospital, Tianjin, China; 3 Department of Pharmacology & Therapeutics, University of Florida College of Medicine, Gainesville, Florida, USA; 4 Diabetes Institute, University of Florida College of Medicine, Gainesville, Florida, USA; 5 Immunobiology and Therapy Unit, Institut Pasteur, Inserm U1224, Université Paris Cité, Paris, France; 6 Departments of Molecular & Integrative Physiology, University of Michigan Medical School, Ann Arbor, Michigan, USA; 7 Departments of Cell and Developmental Biology, University of Michigan Medical School, Ann Arbor, Michigan, USA

**Keywords:** amino acids, phospho-eIF2α, endoplasmic reticulum, preproinsulin, biosynthesis

## Abstract

Insulin is made from proinsulin, but the extent to which fasting/feeding controls the homeostatically regulated proinsulin pool in pancreatic β-cells remains largely unknown. Here, we first examined β-cell lines (INS1E and Min6, which proliferate slowly and are routinely fed fresh medium every 2–3 days) and found that the proinsulin pool size responds to each feeding within 1 to 2 h, affected both by the quantity of fresh nutrients and the frequency with which they are provided. We observed no effect of nutrient feeding on the overall rate of proinsulin turnover as quantified from cycloheximide-chase experiments. We show that nutrient feeding is primarily linked to rapid dephosphorylation of translation initiation factor eIF2α, presaging increased proinsulin levels (and thereafter, insulin levels), followed by its rephosphorylation during the ensuing hours that correspond to a fall in proinsulin levels. The decline of proinsulin levels is blunted by the integrated stress response inhibitor, ISRIB, or by inhibition of eIF2α rephosphorylation with a general control nonderepressible 2 (not PERK) kinase inhibitor. In addition, we demonstrate that amino acids contribute importantly to the proinsulin pool; mass spectrometry shows that β-cells avidly consume extracellular glutamine, serine, and cysteine. Finally, we show that in both rodent and human pancreatic islets, fresh nutrient availability dynamically increases preproinsulin, which can be quantified without pulse-labeling. Thus, the proinsulin available for insulin biosynthesis is rhythmically controlled by fasting/feeding cycles.

During cycles of fasting and feeding, pancreatic β-cells sense changes in the availability of nutrients and adjust insulin release to maintain euglycemia (1). Additionally, changes in nutrient availability (2), especially glucose (3), can acutely regulate the translation of newly synthesized proinsulin in the endoplasmic reticulum (ER) of β-cells (4, 5, 6, 7, 8)—independently of transcriptional regulation (9). Glucose-dependent biosynthesis of proinsulin [the most abundantly translated gene product in β-cells (9)] is thought to be heterogeneous within the β-cell population (10, 11) with the most active biosynthesis occurring in cells that are replenishing the proinsulin pool for the biogenesis of secretory granules [for future cycles of insulin secretion (12)]. Change in nutrient availability (such as occurs during cycles of fasting/feeding) is also reported to modulate proinsulin degradation activity in β-cells (13, 14, 15, 16, 17). Whereas under physiological conditions, mature insulin secretory granules in β-cells have a half-life >1 day (18); the half-life of proinsulin molecules is much shorter (19, 20).

Fasting and feeding does influence the nutrients available to tissues. For example, in wildtype mice, overnight fasting reduces circulating levels of glutamine and serine [and some others, including Ala, Gly, and Lys (21)]. Additional amino acids can be contributed by intracellular proteolytic degradative machineries that include both lysosomal (13, 17) and proteasomal (20) sources. It is likely that intracellular proinsulin degradation and biosynthesis may be metabolically connected, yet we are still left with a limited understanding of which pathway(s) is(are) the rate-controlling contributor(s) to fasting/feeding-dependent regulation of the steady-state proinsulin pool, which is the physiologic basis for the biosynthetic replenishment of insulin secretory granules (22).

In this report, we have used both β-cell lines and primary pancreatic islets to examine the dynamic impact of fasting and feeding on the proinsulin pool size in pancreatic β-cells. The results provide important insight into normal β-cell physiology and may open doors to a deeper understanding of the pathophysiology of β-cell dysfunction in diabetes.

## Results

### Nutrient-dependent fluctuations in proinsulin pool size in pancreatic β-cell lines

β-cells maintain availability of proinsulin for insulin secretory granule homeostasis (12); we seek to understand the rate-controlling event(s) in this homeostatic process. For this, we first sought to examine the proinsulin pool size in the INS1E β-cell line (23) that double every ∼30 h; such cells grow to confluence and are essentially provided “time-restricted” feeding (routinely, they are fed every 2–3 days). Between feedings, β-cells consume nutrients and thus go through cycles of feeding and fasting (24). As a standard protocol, we used 80% confluent cells that were fed exactly 24 h before the start of each experiment.

**Figure 1:**
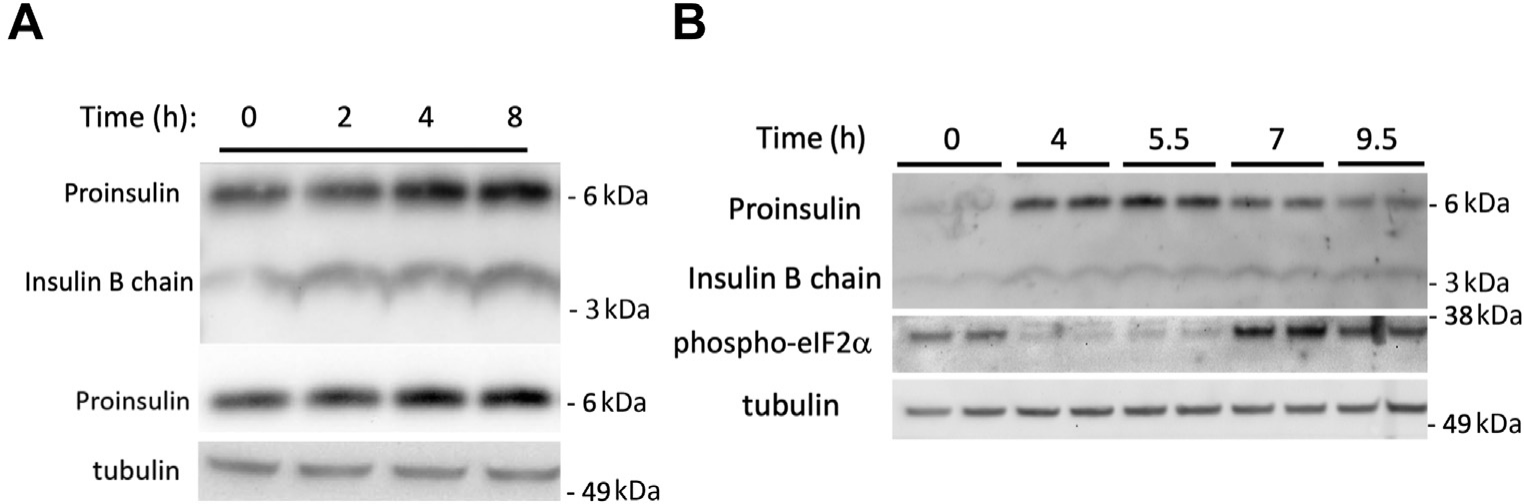
**Nutrient-dependent fluctuation in proinsulin pool size in INS1E β-cells.** Cells grown in RPMI-1640 medium were seeded and grown in tissue culture plates for 2 days, fed every 24 h (100 μl per cm^2^ surface area). *A*, a set of cells were lysed at the “zero time” (24 h after the last feeding), and the other sets were refed complete RPMI medium spiked to 25 mM glucose at (100 μl/cm^2^) before lysis at different times as indicated. Lysates were resolved by reducing SDS-PAGE and analyzed by immunoblotting with guinea pig anti-insulin (*upper panel*), mouse mAb anti-rodent proinsulin (*middle panel*), or tubulin as a loading control (*lower panel*). *B*, a protocol similar to panel A was used with complete medium at the usual RPMI level of 11.1 mM glucose; samples run as biological duplicates. Lysates were resolved by reducing SDS-PAGE and analyzed by immunoblotting with guinea pig anti-insulin (*upper panel*), rabbit polyclonal anti-phospho-eIF2α (*middle panel*), or tubulin as a loading control (*lower panel*). Molecular weight markers (kDa) are indicated.

**Figure 2:**
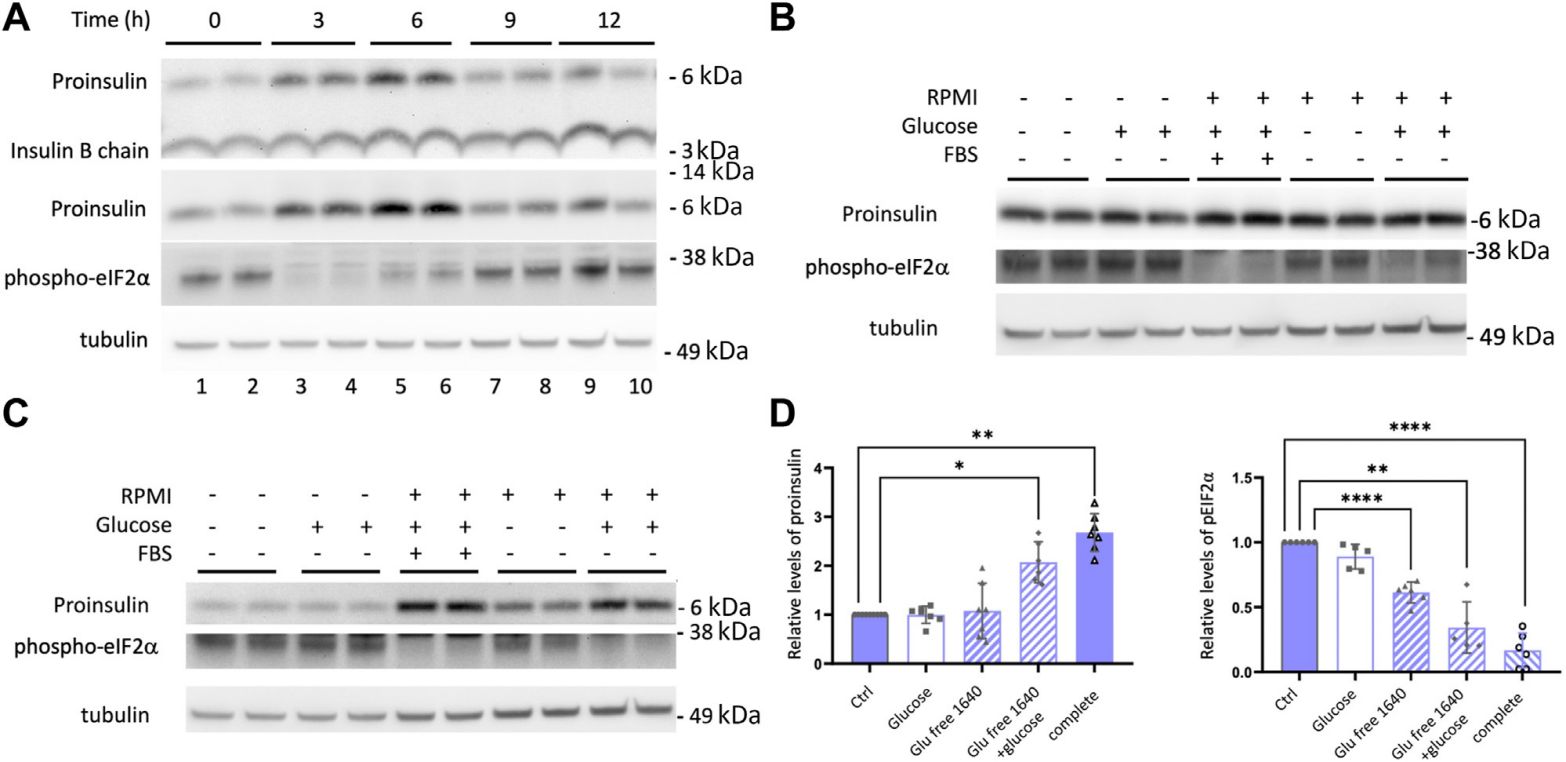
**Contribution of glucose and amino acids to the regulation of proinsulin pool size in INS1E cells.** Cells were seeded and initially fed as in Figure 1. *A*, using a protocol similar to that in Figure 1 with timepoints as indicated: cell lysates (biological duplicates) were analyzed by immunoblotting with guinea pig anti-insulin (*upper panel*), mouse mAb anti-proinsulin (*middle panel*), rabbit polyclonal anti-phospho-eIF2α (third panel), or tubulin as a loading control (*bottom panel*). *B* and *C*, at the start of the experiment, biological duplicates either remained unfed in their 24 h-old medium or were fed selectively with fresh medium components as indicated, and the cells lysed thereafter at 30 min (*B*) or 4 h (*C*). Cell lysates were resolved by reducing SDS-PAGE and analyzed by immunoblotting with mouse mAb anti-rodent proinsulin, rabbit polyclonal anti-phospho-eIF2α, or tubulin as a loading control. Molecular weight markers (kDa) are indicated. *D*, quantitation (mean ± SD) of proinsulin and phospho-eIF2α protein levels relative to that obtained in the unfed control (Ctrl) sample; ∗*p* < 0.05, ∗∗*p* < 0.01, ∗∗∗∗*p* < 0.0001.

In an initial experiment, a set of β-cells were lysed as a measure of the ‘starter’ level of proinsulin, and the remaining wells were fed fresh medium that was “spiked” to a level of 25 mM glucose (rather than the usual 11 mM glucose contained in standard RPMI growth medium). In the ensuing 2, 4, and 8 h after feeding fresh nutrients, proinsulin levels visibly rose, as measured by immunoblotting with an insulin antibody (Fig. 1*A* *upper panel*) and confirmed with a proinsulin-specific mAb (Fig. 1*A* *middle panel*). This result is expected as high glucose is known to induce preproinsulin mRNA translation (12). When this experiment was repeated under standard feeding conditions, proinsulin levels still jumped dramatically at 4 to 5.5 h after feeding but subsided thereafter (Fig. 1*B* *upper panel*). These results were confirmed in multiple independent experiments and validated by immunoblotting with a proinsulin-specific mAb (Fig. 2*A*), indicating that there are actually major nutrient-dependent fluctuations in proinsulin pool size in INS1E cells (quantitation described, below).

In addition to glucose, RPMI growth medium contains amino acids (and vitamins and is supplemented with fetal bovine serum). To examine the role of the various components, we either provided 11.1 mM glucose alone or glucose-free RPMI in cells 24 h after their last feeding. Proinsulin levels did not rise at either 30 min or 4 h after providing fresh glucose alone (Fig. 2, *B* and *C*) nor did we observe a significant increase of proinsulin levels after providing amino acids (present in glucose-free RPMI, Fig. 2, *C* and *D*). However, combining glucose and amino acids resulted in a significant increase in the proinsulin pool size (Fig. 2*C*, *quantified in 2D*), as was also seen in complete medium. These data strongly suggest that amino acids and glucose together contribute to the regulation of proinsulin pool size after feeding.

**Figure 3:**
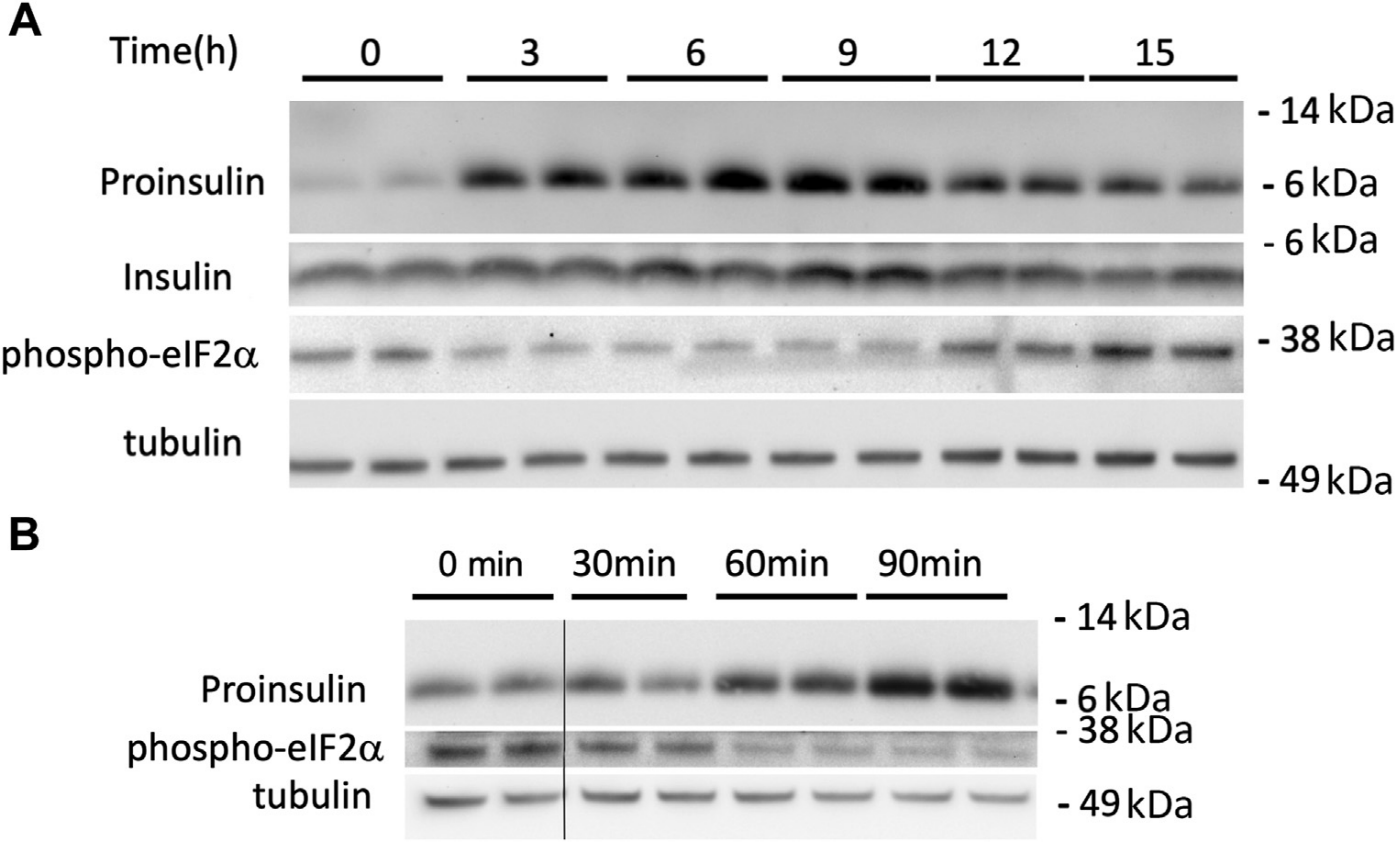
**Nutrient-dependent fluctuation in proinsulin pool size in Min6 β-cells.** Cells grown in DMEM medium (containing 25 mM glucose) were seeded and grown in tissue culture plates for 2 days, fed every 24 h (100 μl per cm^2^ surface area). *A* and *B*, a set of cells were lysed at the “zero time” (24 h after the last feeding), and other sets (biological duplicates) were refed complete DMEM (100 μl/cm^2^) before lysis at different times after feeding. Cell lysates were resolved by reducing SDS-PAGE and analyzed by immunoblotting with mouse mAb anti-rodent proinsulin (*upper panel*), guinea pig anti-insulin (B-chain, second panel), anti-phospho-eIF2α (third panel; there is a gel artifact between the duplicates at 12 h), or tubulin as a loading control (*lower panel*). *A*, cell lysed every 3 h during a 15 h time course. *B*, cells lysed every 30 min during a 90 min time course. The first two lanes are from the same gel and membrane, but the image has been spliced and is separated by a vertical line. Molecular weight markers (kDa) are indicated. DMEM, Dulbecco's modified Eagle's medium.

Min6 β-cells were originally generated by a different protocol [and from a different species (25)] than that of INS1E—they double every ∼48 h and are routinely grown in the presence of 25 mM glucose—yet also retain glucose responsiveness. In culture, these cells also receive time-restricted feeding, every 2 to 3 days. Once again, by immunoblotting, we observed that 24 h after the last feeding of near-confluent cells, a new round of feeding resulted in a prompt boost of the size of the proinsulin pool, seen at 3, 6, and 9 h before subsiding (Fig. 3*A*). Indeed, an acute increase in β-cell proinsulin level after feeding was detectable within 60 to 90 min of medium change (Fig. 3*B*). These data indicate that fresh nutrients contribute to upregulation of the proinsulin pool size in both INS1E and Min6 cells, and this effect occurs rapidly (*i.e.*, 1–2 orders-of-magnitude faster than the proliferation rate of these cells).

### The effect of nutrient availability on proinsulin disappearance in pancreatic β-cell lines

**Figure 4:**
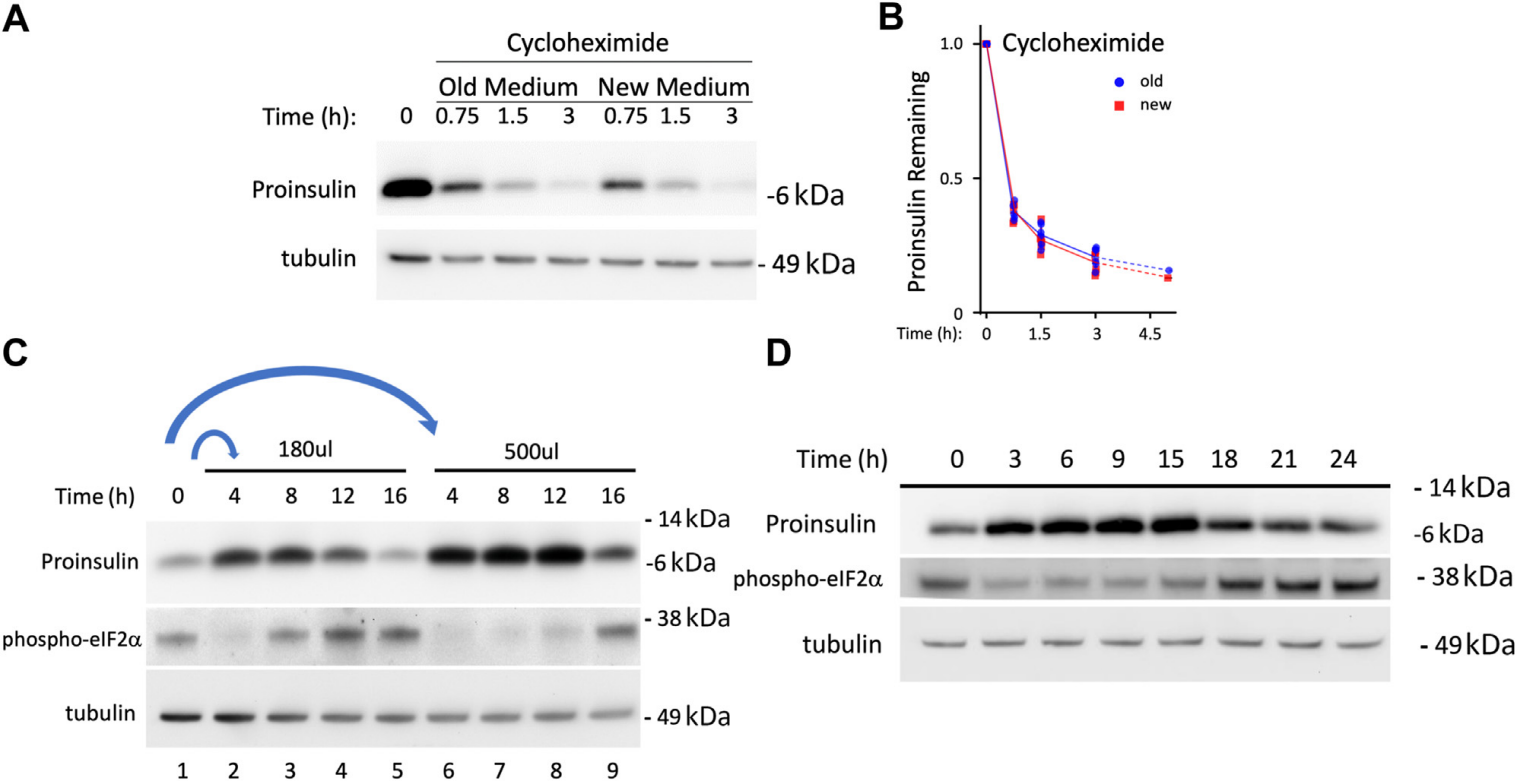
**The effect of nutrient availability on proinsulin disappearance and appearance in pancreatic β-cells.***A*, Min6 cells were seeded and fed exactly as in Figure 3. One set of cells was then lysed (“zero time”). In a second set of samples, old medium was removed, quickly spiked with cycloheximide (100 µg/ml final concentration) and added back to the same cells with subsequent lysis at different times thereafter, as indicated. In a third set of samples, cells were fed new complete medium containing cycloheximide before lysis at different times thereafter, as indicated. Cell lysates were resolved by reducing SDS-PAGE and immunoblotting with mouse mAb anti-rodent proinsulin (*middle panel*; a long exposure is shown to increase detection of proinsulin); tubulin was a loading control (*lower panel*). *B*, quantitation of fraction of proinsulin (normalized to tubulin) remaining at different times after cycloheximide addition (mean ± SD; error bars are included but underlie the data shown), with no significant differences between old and new medium at any time point (n = 5 independent experiments; the 5-h time point was included for only two independent experiments). *C*, INS1E cells grown in RPMI-1640 medium (11.1 mM glucose) were seeded and grown in tissue culture plates for 2 days, fed every 24 h (100 μl per cm^2^ surface area). One set of cells was lysed (“zero time;” 24 h after the last feeding), and the remaining cells were fed either at 180 μl per 2 cm^2^ surface area or 500 μl per 2 cm^2^ surface area of fresh complete RPMI. *D*, Min6 cells grown in DMEM medium (25 mM glucose) were seeded and grown in tissue culture plates for 2 days, fed every 24 h (100 μl per cm^2^ surface area). In contrast with Figure 3*A*, cells were fed with the larger volume of 500 μl per 2 cm^2^ surface area of fresh complete DMEM. In both (*C* and *D*), the cells were lysed at various time points as indicated. Lysates were resolved by reducing SDS-PAGE and immunoblotting with mouse mAb anti-rodent proinsulin (*upper panel*), anti-phospho-eIF2α (*middle panel*), or tubulin as a loading control (*lower panel*). Molecular weight markers (kDa) are indicated. DMEM, Dulbecco's modified Eagle's medium.

Potentially, the change in proinsulin pool size in β-cells might be explained by enhanced proinsulin turnover when fresh nutrients are unavailable. Proinsulin disappearance within pancreatic β-cells can occur from multiple pathways: secretion of unprocessed proinsulin, endoproteolytic conversion to insulin, degradation *via* immature secretory granule fusion with lysosomes, autophagic degradation of Golgi-derived, and/or post-Golgi–derived proinsulin, as well as ER-degradative events such as ER-associated degradation and ER-phagy. While it is difficult to experimentally quantify proinsulin turnover within each individual pathway concurrently, a ‘cycloheximide-chase’ protocol does allow for examination of the cumulative impact on proinsulin turnover from all such pathways combined. At 24 h after feeding from one set of samples, we removed the “old” spent medium, spiked that medium with cycloheximide (CHX), and returned it back to the same cells for varying periods of time. In a second set of samples, we refed the cells with “new” fresh medium that was similarly spiked with CHX. By immunoblotting with anti-proinsulin, we noted that the overall rate of proinsulin disappearance was unaffected in cultured β-cells bathed in old medium compared to those bathed in new medium (Fig. 4*A* *quantified in B*). Thus, the effect of nutrient availability to cause dramatic and rapid changes in proinsulin pool size cannot be explained by proinsulin turnover.

### Nutrient regulation of proinsulin pool size involves integrated stress response signaling

In the foregoing experiments in β-cell lines, we repeatedly noted diminished phosphorylation of eIF2α shortly after feeding fresh nutrients to β-cells (Figure 1, Figure 2*A*, and 3*A*), and this was apparent within 30 min of feeding INS1E cells (Fig. 2*B*) or Min6 cells (Fig. 3*B*) and persisted for several hours after feeding (Figs. 2*C* and *D*, and 3*A*). Regulation of phospho-eIF2α levels is a central feature of the classic integrated stress response (ISR) (26), and it is known that in β-cells, stimulatory glucose is one of the factors that can promote diminished levels of phospho-eIF2α (27). However, there was no significant effect on phospho-eIF2α levels at 4 h after feeding 11.1 mM glucose alone, whereas amino acids alone did promote diminished phospho-eIF2α (as did the two groups of nutrients together, Fig. 2*D*).

We noted that whereas the peak of proinsulin levels (Figure 1, Figure 3*A*, and 2, *A* and *C*) was accompanied by diminished phosphorylation of eIF2α, there was a subsequent decline of proinsulin levels that accompanied a rebound of phospho-eIF2α (Figure 1, Figure 2*A*, and 3*A*). This rephosphorylation indicates activity of one or more eIF2α kinases linked to the ISR (26, 28). Of the potential stresses that may activate one or more of these kinases, one possible basis for eIF2α rephosphorylation could be prior ER accumulation of proinsulin triggering activation of PERK (29). Indeed, the increased β-cell proinsulin pool after feeding was accompanied by a parallel increase in misfolded proinsulin engaged in intermolecular disulfide-linked complexes (Fig. S1). An alternative explanation for the rephosphorylation of eIF2α might involve the consumption of amino acids, with accumulation of uncharged tRNAs that activate general control nonderepressible 2 (GCN2) (30, 31). We reasoned that if rephosphorylation of eIF2α resulted from prior ER proinsulin accumulation, then this would be *accentuated* if there were even greater levels of proinsulin accumulation as a result of increased nutrient availability, whereas if eIF2α rephosphorylation resulted from depletion of amino acids (thereby activating GCN2), then such rephosphorylation should be *suppressed* by increasing amino acids made available at the time of feeding.

Indeed, simply increasing the feeding volume of INS1E cells increased proinsulin protein more robustly yet suppressed eIF2α for a longer interval (Fig. 4*C*)—extending peak proinsulin levels to a later time. This extended peak that accompanied sustained suppression of phospho-eIF2α was also observed in Min6 cells fed with a larger volume of fresh medium (Fig. 4*D*) rather than a smaller volume (Fig. 3*A*). The data imply that the ISR activation occurring many hours after the last feeding of β-cells may be a response to nutrient depletion.

### Nutrient-depleted upregulation of phospho-eIF2α in β-cells

**Figure 5:**
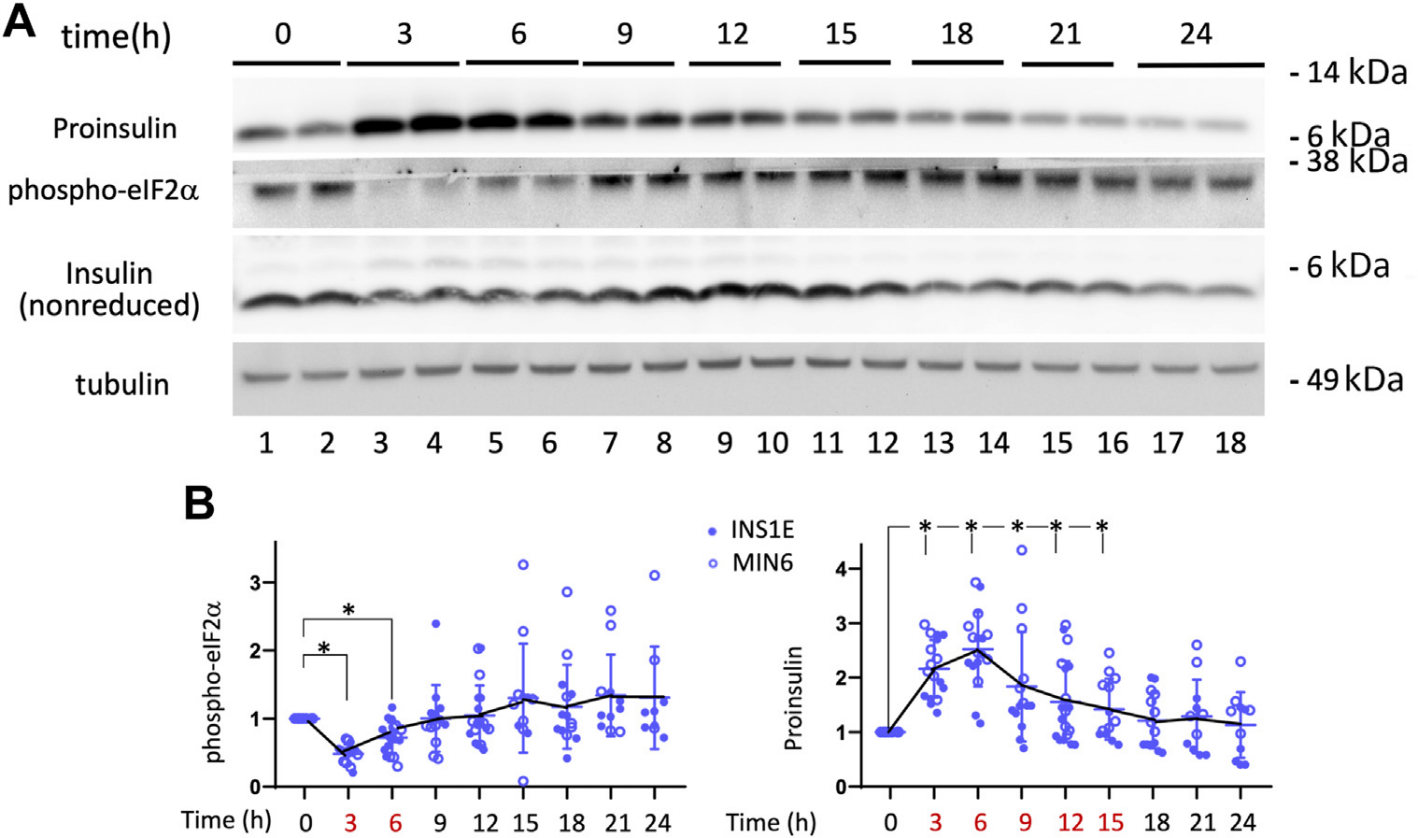
**The time course of proinsulin, insulin, and phospho-eIF2α in pancreatic β-cells.***A*, INS1E cells grown in RPMI-1640 medium were seeded and grown in tissue culture plates for 2 days, fed every 24 h (100 μl per cm^2^ surface area). A set of cells were lysed at the “zero time” (24 h after the last feeding), and the other sets were refed complete RPMI medium (100 μl/cm^2^) before lysis at different times over a 24 h time course. Lysates were resolved by reducing SDS-PAGE and analyzed by immunoblotting with mouse mAb anti-rodent proinsulin (*upper panel*), anti-phospho-eIF2α (*second panel*), or tubulin as a loading control (*bottom panel*), or resolved by nonreducing SDS-PAGE for immunoblotting with guinea pig anti-insulin (third panel). Molecular weight markers (kDa) are indicated. *B*, using the protocol described in panel A, quantitation (mean ± SD) of phospho-eIF2α and proinsulin protein levels (normalized to tubulin) from both INS1E cells (RPMI medium with 11.1 mM glucose, *solid dots*) and Min6 cells in (DMEM with 25 mM glucose, *open dots*) are shown. ∗*p* < 0.05 compared to the zero-time point. DMEM, Dulbecco's modified Eagle's medium.

Even in timed-feeding experiments that provide greater initial nutrients, proinsulin levels eventually fell toward their starting levels, accompanied by eIF2α rephosphorylation in INS1E (Fig. 3*A*) and Min6 cells (Fig. 4*D*). In β-cell lines, it was notable that a wave of increased proinsulin at 3 to 6 h postfeeding (Fig. 5*A* *lanes 3–6*) including both properly and improperly folded proinsulin (Fig. S2) was followed thereafter by increased mature insulin, which peaked ∼12 h postfeeding (Fig. 5*A* *lanes 9 + 10*). However the increase is self-limited because after the proinsulin pool size has risen by ∼250% at 6 h post-timed-feeding, at that point, eIF2α phosphorylation has begun to rebound (Fig. 5*B*).

**Figure 6:**
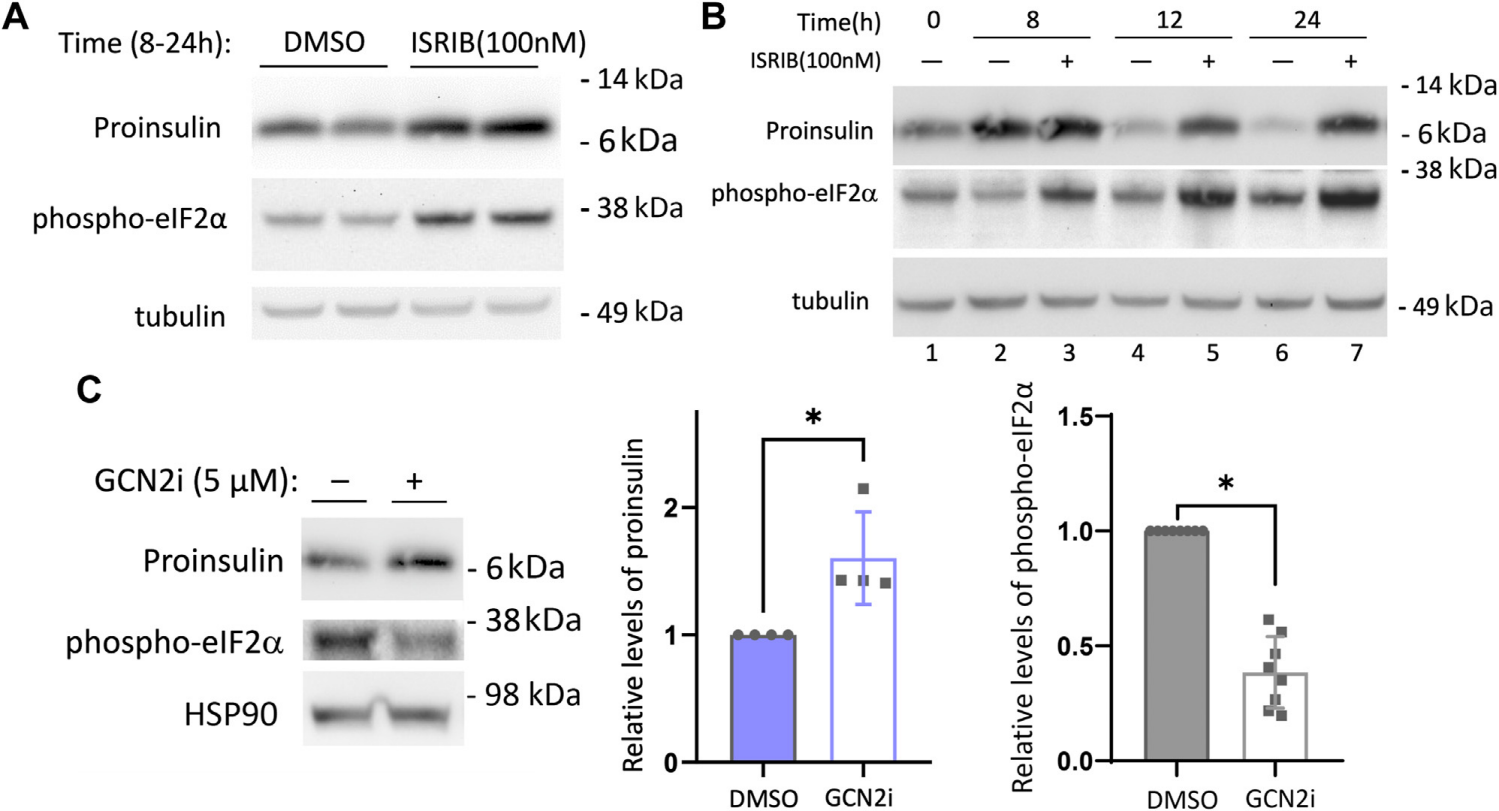
**Nutrient-depleted upregulation of phospho-eIF2α in β-cells.***A* and *B*, INS1E cells grown in RPMI-1640 medium were seeded and grown in tissue culture plates for 2 days, fed every 24 h (100 μl per cm^2^ surface area). *A*, At 8 h after the last refeeding, either vehicle (DMSO) or ISRIB (100 nM) was swirled directly into the existing medium, and the cells lysed at the 24 h time point. *B*, At 4 h after the last refeeding, either vehicle (DMSO) or ISRIB (100 nM) was swirled directly into the existing medium, and the cells lysed at the time points indicated. Lysates were resolved by reducing SDS-PAGE and analyzed by immunoblotting with mouse mAb anti-rodent proinsulin (*upper panel*), anti-phospho-eIF2α (*middle panel*), or tubulin as a loading control (*bottom panel*). *C*, Min6 cells grown in complete DMEM medium (25 mM glucose) were seeded and grown in tissue culture plates for 2 days, fed every 24 h (100 μl per cm^2^ surface area). At 8 h after the last refeeding, either vehicle or GCN2 inhibitor (GCN2i, 5 μM) was swirled directly into the existing medium and the incubation continued for another 2 h before cell lysis. At *left*: Lysates were resolved by reducing SDS-PAGE and analyzed by immunoblotting with mouse mAb anti-rodent proinsulin (*upper panel*) and anti-phospho-eIF2α (second panel); HSP90 (*bottom panel*) is a loading control. Molecular weight markers (kDa) are indicated. At right: Quantitation (mean ± SD) of the levels of proinsulin and phospho-eIF2α (normalized to HSP90), respectively. ∗*p* < 0.05 compared to vehicle alone. DMEM, Dulbecco's modified Eagle's medium; GCN2, general control nonderepressible 2.

To establish that the increase of phospho-eIF2α several hours after feeding is not only correlative but actually regulates the decline of β-cell proinsulin levels, beginning at 8 h after feeding INS1E cells, we added the ISR inhibitor ISRIB and continued to 24 h. ISRIB inhibits the action of phospho-eIF2α on eIF2B and [despite resulting in increased phospho-eIF2α levels, as expected (28, 32)]. ISRIB addition was accompanied by an increase of proinsulin pool size (Fig. 6*A* scanned image data in Fig. S3*A*). With ISRIB addition beginning 4 h after feeding (when eIF2α phosphorylation is lower than starting levels), the change in proinsulin pool size was small at the next measured time point (8 h), whereas if ISRIB was continued until 12 h or 24 h postfeeding, the fold-increase of proinsulin grew larger (a time when proinsulin steady-state levels would otherwise be low; Fig. 6*B* scanned image data in Fig. S3*B*). These data indicate that it is more than a correlation; rather, the bioactivity of phospho-eIF2α (inhibited by ISRIB) is an important negative regulator of proinsulin pool size as nutrients become depleted. Moreover, addition of GCN2 inhibitor (33) beginning 8 h after feeding lowered phospho-eIF2α levels while raising proinsulin levels (Fig. 6*C*).

**Figure 7:**
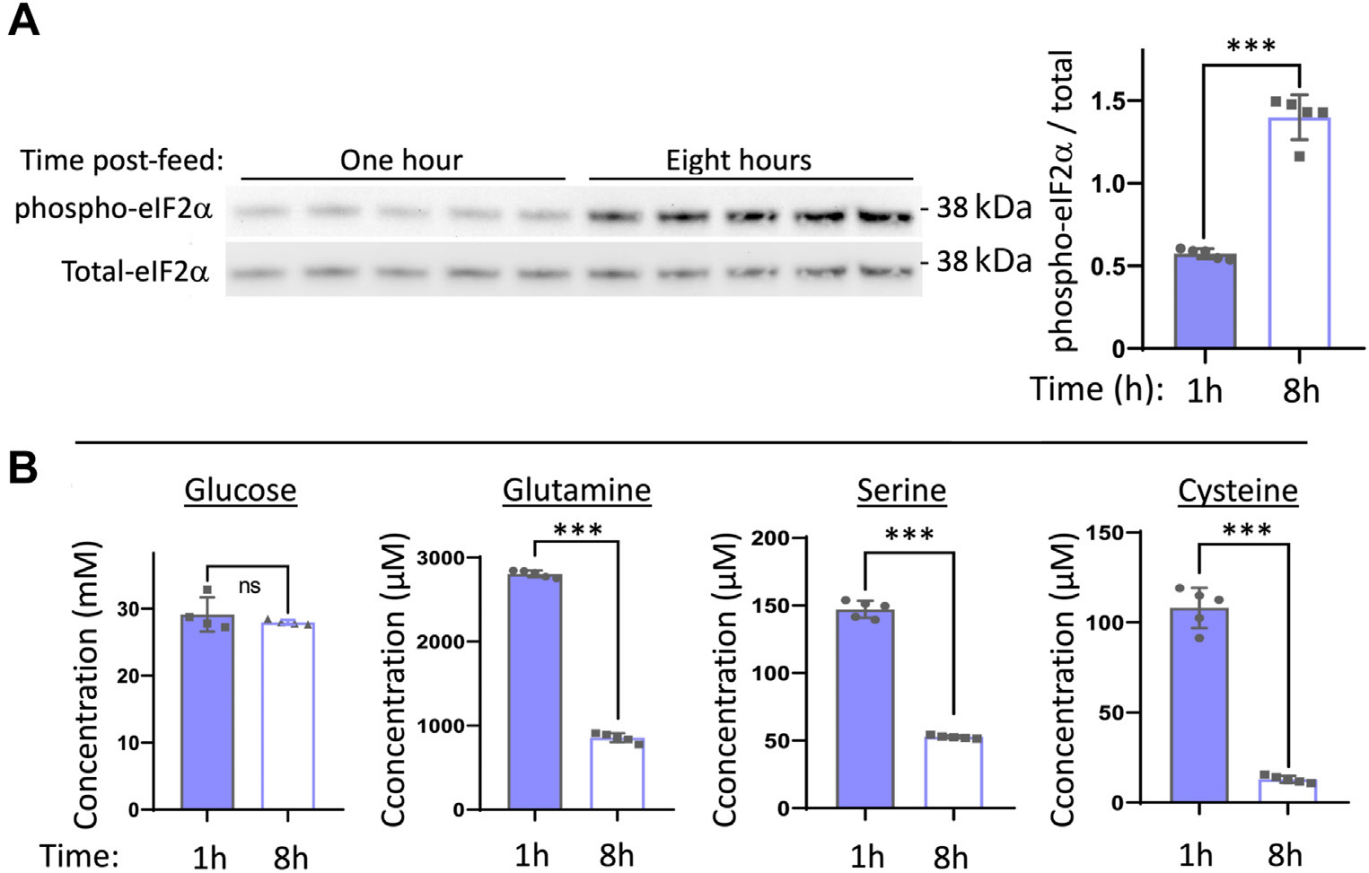
**Min6 β-cells selectively consume amino acids after feeding.** Min6 cells grown in complete DMEM medium (25 mM glucose) were seeded and grown in tissue culture plates for 2 days, fed every 24 h (100 μl per cm^2^ surface area). At 1 h or 8 h after the last refeeding (as indicated), the media were collected, and cells were lysed. *A*, phospho-eIF2α and total eIF2α levels in cell lysates [molecular weight markers (kDa) are indicated], quantified at right (mean ± SD; ∗∗∗*p* < 0.001). *B*, glucose concentration was measured by glucometer, whereas the absolute concentration of all 20 amino acids in the collected media were measured by LC-MS (including calibrated standards). Seventeen of these amino acids showed no decrease; three amino acids declined precipitously, as shown. n = 5 biological replicates (mean ± SD; ∗∗∗*p* < 0.001). DMEM, Dulbecco's modified Eagle's medium.

In these Min6 cells in the fasted state, GCN2 rather than PERK provides the kinase activity that exerts the greatest impact on phospho-eIF2α levels (Fig. S3*C*). As GCN2 is activated by amino acid depletion, we collected medium at 1 h and 8 h after a timed-feeding—the latter of which is a time when phospho-eIF2α levels have increased significantly (Fig. 7*A*). Media glucose levels at the 8 h time point remained at >95% of the 1 h value (Fig. 7*B* left). We used LC-MS to analyze consumption of amino acids from the medium by the β-cells. We observed no decrease in the medium for 17 of the 20 amino acids, but a marked decline in the levels of glutamine, serine, and cysteine (Fig. 7*B*). Altogether, these data support that β-cells sense fasting, and during this period may activate the amino acid depletion response through GCN2.

### Direct evidence that nutrient-dependent upregulation of the proinsulin pool is biosynthetic

It is widely recognized that nutrient fluctuations induce catabolic as well as anabolic responses (34). Beyond results from our ‘cycloheximide-chase’ experiments (Fig. 4), we sought independent direct evidence in β-cells that the fresh nutrient-dependent increase of proinsulin pool size reflects an increase in proinsulin biosynthesis. Biosynthesis of new proinsulin is traditionally measured by pulse-labeling radiolabeled amino acids, but unfortunately at different times after feeding, isotopic labeling efficiency changes (*i.e.*, when the bathing medium has diminished unlabeled cysteine, the specific radioactivity of labeled cysteine is altered). However, as the preproinsulin half-life is ∼1 min (35), it should be an ideal marker of biosynthesis from the Ins gene(s), with the understanding that successful translocation into the ER causes immediate conversion to proinsulin (36).

**Figure 8:**
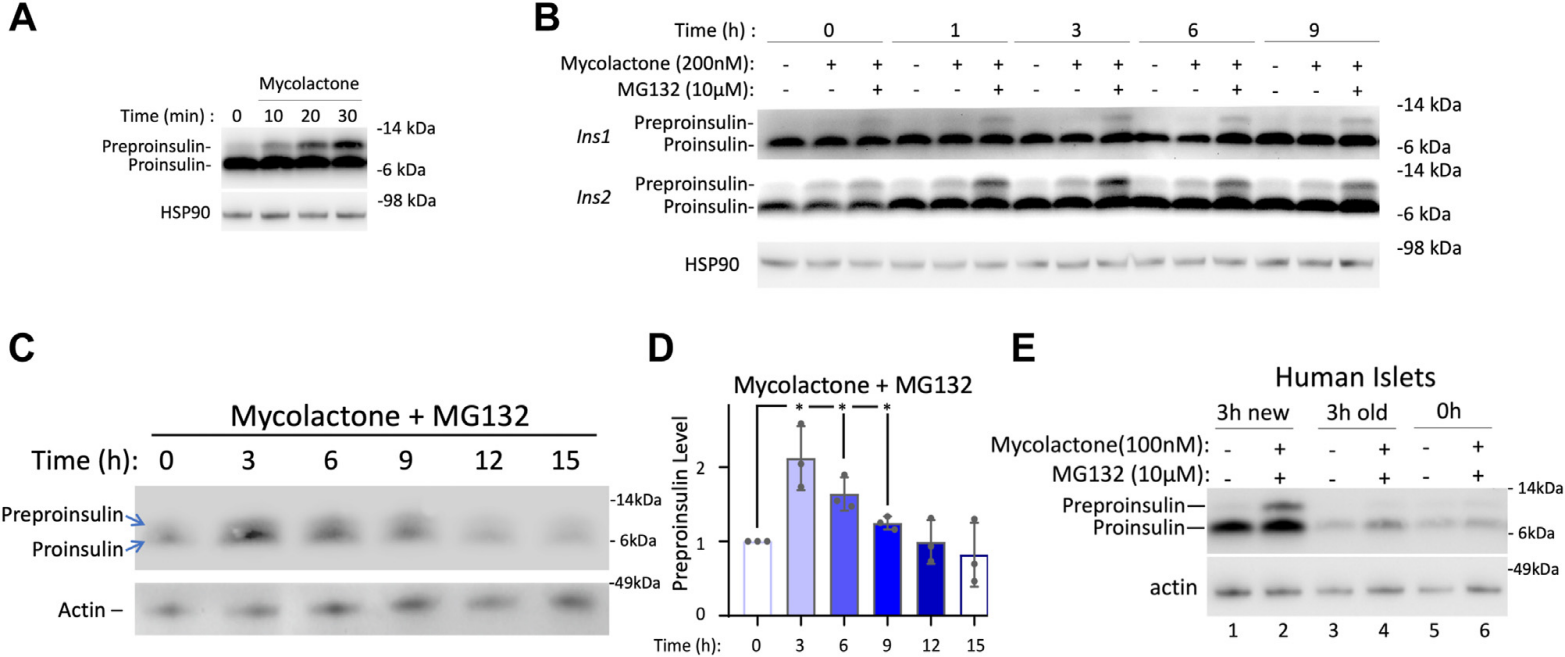
**Direct evidence that nutrient-dependent upregulation of the proinsulin pool is biosynthetic.***A* and *B*, Min6 cells grown in complete DMEM medium (25 mM glucose) were seeded and grown in tissue culture plates for 2 days, fed every 24 h (100 μl per cm^2^ surface area) before initiating this experiment by refeeding the cells (100 μl/cm^2^) for 4 h. *A*, mycolactone (200 nM) was added for the time points indicated (up to 30 min), and the cells lysed, resolved by reducing SDS-PAGE, and analyzed by immunoblotting with anti-rodent proinsulin; HSP90 is a loading control. *B*, Min6 cells were treated as in Figure 3*A*, but during the last 30 min before each time point, the cells were treated with either vehicle alone, mycolactone (200 nM), or mycolactone plus MG132 (10 μM). Lysates were resolved by reducing SDS-PAGE and analyzed by immunoblotting with mAb anti-rodent proinsulin that favors the Ins1 gene product and polyclonal anti-rodent proinsulin that favors the Ins2 gene product; these antibodies also recognize preproinsulin as indicated. HSP90 (*middle panel*) is a loading control. *C*, murine islets were incubated in a limiting volume (2 μl/islet) of complete RPMI-1640 (11.1 mM glucose) for 24 h and then the medium spiked for 30 min with mycolactone (100 nM) plus MG132 (10 μM) (‘zero time point’), or islets were refed fresh medium for the times indicated, and during the last 30 min before each time point, the islets were treated with mycolactone plus MG132. Cell lysates were resolved by reducing SDS-PAGE and immunoblotting with rabbit polyclonal anti-proinsulin as in panel B; actin (*bottom panel*) is a loading control. *D*, quantitation of preproinsulin as a function of time after feeding (normalized to actin; the preproinsulin level at the zero-time point was set to 1.0 in each of three independent experiments; mean ± SD; ∗*p* < 0.05). *E*, human islets (from a nondiabetic donor) were incubated in a limiting volume (2 μl/islet) of Prodo medium (5.6 mM glucose) for 24 h. One set of islet samples was then either untreated (vehicle alone) or treated with mycolactone (100 nM) + MG132 (10 μM) for 30 min before lysis (“zero time”, lanes 5 + 6). In a second set of islet samples, the old medium was removed and returned back to the same islets for 3 h plus 30 min in the presence of either vehicle or mycolactone + MG132 (lanes 3 + 4). In a third set of islet samples, the old medium was removed and replaced with new fresh medium for 3 h plus 30 min in the presence of either vehicle or mycolactone+MG132 (lanes 1 + 2). Islet lysates were resolved by reducing SDS-PAGE and analyzed by immunoblotting with anti-human-proinsulin (also recognizing preproinsulin as indicated); actin is a loading control. Molecular weight markers (kDa) are indicated. DMEM, Dulbecco's modified Eagle's medium.

First, we examined preproinsulin levels by immunoblotting after addition of mycolactone, an inhibitor of the Sec61 translocon (37). Inhibiting protein translocation over 30 min resulted in an increase of preproinsulin levels (Fig. 8*A*). However, untranslocated preproinsulin is susceptible to proteasomal disposal (20, 38, 39); therefore, we examined preproinsulin levels in Min6 cells at different times after feeding, but during the last 30 min we added both mycolactone and MG132 (proteasome inhibitor). This maneuver made it obvious that preproinsulin (1 + 2) levels rise in the early hours after feeding and begin to gently decline thereafter (Fig. 8*B*). We also observed that the ratio of ribosomal 18S RNA: Ins1 + 2 mRNA from ‘RiboTagged’ (RPL22-Flag) ribosomes in Min6 cells rose shortly after feeding—suggestive of an increase of Ins1 + 2 polysomes (Fig. S4).

We then turned attention to murine (and human) pancreatic islets. We incubated isolated islets for 24 h in a limiting quantity of nutrient-rich “islet recovery medium” (*i.e.*, 30 islets in 60 μl). Murine islets were then either lysed (“time zero”) or transferred to fresh RPMI medium for varying times; during the last 30 min, we added mycolactone + MG132. We observed a clear increase in preproinsulin (as well as proinsulin) that peaked after providing fresh nutrients and subsequently declined (Fig. 8*C*, quantified in 8D). In human islets, when mycolactone + MG132 were added for 30 min at 24 h postfeeding, there was little change in preproinsulin/proinsulin (Fig. 8*E* lanes 5 + 6). When ‘old’ medium was removed and returned back to the same islets (for 3 h plus 30 min with mycolactone + MG132), there was still only a very modest change in preproinsulin/proinsulin (Fig. 8*E* lanes 3 + 4). However, when fresh nutrient-containing medium was provided (plus mycolactone + MG132 for 30 min), there was a robust increase in proinsulin (from the first 3 h; Fig. 8*E* lane 1) plus an obvious increase of preproinsulin (from the final 30 min with mycolactone + MG132), reflecting brisk nutrient-dependent proinsulin biosynthesis (Fig. 8*E* lane 2). Altogether, these independent lines of evidence demonstrate a nutrient-dependent increase of the proinsulin pool in β-cell lines, rodent islets, and human islets, primarily involving a biosynthetic response.

## Discussion

Cyclical circadian rhythms influence mammalian metabolism and behavior, including fasting/feeding cycles that affect glucose homeostasis (40). Indeed, islet β-cells exhibit protein oscillations linked to regulators such as CLOCK and BMAL1 (41, 42). Within the overall circadian cycle, feeding drives transcriptional and translational activities in β-cells and other cell types (43, 44, 45).

The size of the proinsulin pool (controlled both by proinsulin turnover and synthesis) is a central factor in homeostatically maintaining the insulin secretory granule population (22). We are not aware of systematic studies of the ‘steady-state proinsulin pool size’ in response to normal physiological cues. Ideally, we would like to know the proinsulin-preproinsulin pool size within islets of living animals/humans across an entire 24 h time-restricted feeding cycle (40). However, islet isolation takes several hours, with major changes of the nutrient environment. Here we have recapitulated time-restricted nutrient exposure for β-cells in culture and for isolated murine or human islets. The results appear remarkably consistent across the different systems and lead to the conclusion that time-restricted feeding triggers a rapid upward wave in the size of the proinsulin pool, which can be used for making new insulin in the ensuing hours.

Nutrient depletion can activate protein catabolism, and it seemed quite possible that fasting-dependent changes in the proinsulin pool might be largely regulated by accelerated proinsulin disappearance from β-cells. However, our data point away from this; instead, several independent lines of evidence indicate that nutrient-dependent regulation of the β-cell proinsulin pool is tightly linked to proinsulin biosynthesis. First, acute nutrient exposure leads to a rapid decrease in β-cell phospho-eIF2α levels, which closely correlate with a rise in proinsulin; subsequent nutrient consumption hours later leads to a rebound of phospho-eIF2α that correlates with a decline in proinsulin. Interestingly, the rebound of phospho-eIF2α is delayed when a more nutrient is made available at the outset of the experiment, suggesting that the rebound of phospho-eIF2α is linked to the time needed for nutrient consumption/depletion rather than the prior nutrient-dependent accumulation of proinsulin. Significantly, the fasting-related rebound of phospho-eIF2α is not merely correlative with the suppression of the β-cell proinsulin pool. Rather, the fasting-related decline in proinsulin is very much abated by treating β-cells with ISRIB—a drug that inhibits the bioactivity of phospho-eIF2α (26, 28). Moreover, the rebound of phospho-eIF2α is specifically blunted by inhibition of GCN2 (not PERK). Furthermore, a medium of amino acids containing no glucose yields a greater increase in proinsulin pool size than that observed upon feeding 11.1 mM glucose without amino acids (together, both groups of nutrients yield a near-maximal response).

As noted above, fasting of wildtype mice decreases the circulating levels of glutamine and serine [and some others (21)]. In Min6 β-cells, soon after feeding, we observed little change in extracellular glucose but a major (and selective) depletion of extracellular glutamine and serine (and cysteine). These changes are likely to account for the activation of GCN2, but more studies will be needed to examine cytoplasmic levels of these amino acids during fasting and the ability of repletion with individual amino acids to suppress the rebound of β-cell phospho-eIF2α during fasting.

Our data from cycloheximide-chase experiments do not support that β-cell fasting changes the overall turnover of proinsulin. However, our data should not be taken to mean that nutrient depletion has no impact on proinsulin trafficking that might alter its disappearance *via* secretion, or conversion to insulin (46), or degradation *via* ‘SINGD’ (13), or from post-ER autophagic degradation (47), or *via* ER-associated degradation (16), or ER-phagy (48). Nevertheless, the net effect of all of these pathways combined appears unaffected by the presence or absence of fresh nutrients, whereas time-restricted feeding is obviously linked to the generation of new preproinsulin. To our knowledge, this is the first time that determining preproinsulin abundance (which, for optimal measurement, requires short-term addition of an ER translocation blocker and proteasome inhibitor) has been considered as a means to assess proinsulin biosynthesis. We believe this SDS-PAGE/immunoblotting assay is an extremely valuable tool that will enable the field to explore physiological and pathophysiological changes that may occur in either murine or human islets; of course, the use of translocation and proteasome inhibitors needs to be optimized for dose and time of exposure as we have done here.

There are many reports of experiments on β-cell lines (and islets) designed to examine the effects of various perturbants, or potential therapies, on the biosynthesis of newly synthesized proinsulin and insulin. One variable that has generally not been taken into account is experimental consistency regarding the last time the islets or β-cells were provided fresh nutrients before the experiments were performed. Random times of cell lysis relative to the last feeding can generate remarkable variability in the outcomes of such experiments. Here we have observed consistent and rapid changes in proinsulin pool size after providing fresh nutrients to β-cells or islets; thus in future experiments intended to focus in this area, this parameter needs to be controlled for.

Finally, we note that an increased rate of proinsulin biosynthesis is clearly linked to an increased abundance of misfolded proinsulin in the ER. Nevertheless, our evidence indicates that time-restricted feeding of β-cells triggers a rapid decrease in phospho-eIF2α, suggesting that the misfolded proinsulin triggered by stimulated synthesis from physiological nutrients is not itself sufficient to result in a major degree of PERK activation. Instead, proinsulin biosynthesis is sustained for a number of hours with proinsulin levels growing toward a physiologic maximum (including misfolded proinsulin) before the β-cells experience limitations in amino acid availability that lead to a decline of further proinsulin biosynthesis. However, to the best of our knowledge, no studies have yet been done to examine feeding/fasting-dependent regulation of the proinsulin pool size in the islets of insulin-resistant type 2 diabetic patients or animal models. It seems likely that this area will need to be addressed in order to better understand the β-cell ER stress of type 2 diabetes.

## Experimental procedures

### Antibodies and reagents

Antibodies included guinea pig anti-insulin (Covance, RRID:AB_10013624), mouse mAb anti-rodent proinsulin (Novus Biologicals, RRID:AB_1107982), mouse mAb anti-human proinsulin B-C junction sequence KTRREAEDLQ (Abmart, RRID:AB_2921300), a custom-made rabbit polyclonal antibody against mouse C-peptide-2 (C-PQVAQLELGGGPGAGDLQT, Abmart, RRID:AB_2921302), mouse mAb anti-α-tubulin (Sigma-Aldrich, RRID:AB_477579), mouse mAb anti-beta actin (Proteintech, RRID:AB_2919667), rabbit mAb anti-HSP90 (Cell Signaling, RRID:AB_2233307), and rabbit anti-phospho-eIF2α (Ser51) (Cell Signaling, RRID:AB_330951). All tissue culture reagents were from Invitrogen. Dithiothreitol, MG132, and CHX were from Sigma. GCN2 inhibitor was from MedChemExpress (HY-112654). Mycolactone was isolated from *Mycobacterium ulcerans*, as described (49). RNeasy Plus Mini Kit was from Qiagen (cat. #74134). Anti-flag magnetic agarose beads were from Fisher Scientific.

### Cell culture

Min6 cells were cultured in Dulbecco's modified Eagle's medium (DMEM) medium (25 mM glucose) supplemented with 10% FBS, 0.1 mM β-mercaptoethanol, and penicillin–streptomycin. INS1E cells were cultured in RPMI medium (11 mM glucose) supplemented with 10% FBS, 1 m sodium pyruvate, 0.05 mM β-mercaptoethanol, 10 mM Hepes pH 7.35, and penicillin/streptomycin.

### Mouse islets

Islets from C57BL6/J mice were isolated by pancreatic digestion with collagenase, washing, and centrifugation on Histopaque gradients (cat. #1077, Sigma–Aldrich). The use of mice for this purpose was approved by the University of Michigan Institutional Animal Care and Use Committee (PRO00009936). Before each experiment, isolated islets were washed, handpicked, and incubated in a limiting volume of complete RPMI 1640 medium (bearing 11.1 mM glucose) plus 10% FBS at 37 °C for 24 h. Murine islets were then transferred to fresh complete medium for varying times.

### Human islets

Isolated human islets from a nondiabetic human donor were obtained from Prodo Labs. Islets were washed, handpicked, and incubated in a limiting volume of complete Prodo medium (5.5 mM glucose) for 24 h. Human islets were then further incubated or transferred to fresh complete Prodo medium, as indicated.

### Western blotting

Cells or islets were lysed in RIPA buffer with protease inhibitor cocktail for 15 min (4 °C) and clarified by centrifugation for 15 min @ 12,000 rpm. Before electrophoresis, lysates were boiled in gel sample buffer containing 100 mM dithiothreitol for 5 min and resolved on 4 to 12% gradient NuPage gels, electrotransferred to nitrocellulose, blotted with primary antibodies (1:1000, diluted in TBST with 5% BSA) at 4 °C overnight, incubated with HRP-conjugated-secondary antibody (1:5000) at room temperature for 1 h, with imaging using Clarity Western ECL Substrate (Bio-Rad).

### Amino acid analysis by liquid chromatography-mass spectrometry (LC-MS)

Media samples were analyzed by LC-MS based on the method of Liu *et al.* (50). A 6-point calibration curve was prepared using a mix of unlabeled amino acids (Sigma part AAS18 supplemented with L-asparagine, L-glutamine, and L-tryptophan) with the same internal standard-to-solvent ratio as for the samples. LC-MS used an Agilent 1200 liquid chromatograph coupled to an Agilent 6410 tandem quadrupole mass spectrometer. Amino acid concentration was quantified using Agilent MassHunter Analysis software, measuring peak area ratio to the matching calibrated internal standard.

### Ribosome pull-down assay

Stably transfected (G418-resistant) clone of ‘ribo-tagged’ Min6 cells were generated (Ins2 promoter driving expression of RPL22-Flag), grown in complete DMEM. Immunoprecipitation with anti-Flag was used for pull-down of ribosomes and associated RNAs as described (Fig. S4).

### Statistical analysis

Statistical analysis was assessed by Student’s *t* test and one-way ANOVA test to determine the differences between groups using GraphPad Prism 8 software. Data are presented as means ± SD. *p*-value < 0.05 was considered as statistically significant.

## Data availability

All data are contained within the manuscript, with primary data available upon request (Dr Peter Arvan, University of Michigan, email: parvan@umich.edu).

## Supporting information

This article contains supporting information.

## Conflict of interest

The authors declare that they have no conflicts of interest with the content of this article.
