# Supplementary material for: Nutrient-dependent regulation of β-cell proinsulin content
Source: J Biol Chem. 2023 May 19;299(7):104836. doi: 10.1016/j.jbc.2023.104836 (PMC10302188; doi:10.1016/j.jbc.2023.104836)
Supplement: Supporting Figures S1–S4 [file mmc1.pdf]

Supplemental Figure Legends

Supplemental Figure S1. Identical samples from Fig. 3A (Min6 β-cells) analyzed by immunoblotting for rodent proinsulin under nonreducing conditions.

Supplemental Figure S2. Identical samples from Fig. 5A (INS1E β-cells) analyzed by immunoblotting for proinsulin under nonreducing conditions.

Supplemental Figure S3. A. The bands of Fig. 6A were scanned and quantitated. **B.** The bands of Fig. 6B were scanned and quantitated; the ratio of proinsulin level in the presence of ISRIB to that in the absence of ISRIB is shown for each time point. **C.** An experimental protocol like that of Fig. 6C was performed but comparing the effect of PERK inhibitor to that of GCN2 inhibitor on the level of phospho-eIF2α; tubulin is a loading control. The graph shows phospho-eIF2α levels (normalized to tubulin) from five replicate experiments.

Supplemental Figure S4. Use of Ribo-tagged Min6 cells to examine ribosome engagement of Insulin mRNA. Ribo-tagged Min6 cells in complete DMEM medium (25 mM glucose) were grown in tissue culture plates. After 24 h, media was removed and the cells were re-fed (100 µL per cm^2 surface area) complete DMEM and at the various times indicated, the cells were lysed in 500 µL lysis buffer (1% NP40, 5% glycerol, 1 mM DTT, protease inhibitor cocktail, 200 U/mL RNasin, 100 µg/mL cycloheximide, 50 mM Tris-HCl, 150 mM KCl, 12 mM $MgCl_2$). Cells were not pre-treated with cycloheximide before lysis. Cell lysates were briefly clarified by centrifugation (microfuge, 12,000 rpm) and the supernates containing RPL22-Flag-tagged ribosomes were incubated with anti-Flag magnetic beads on a rotator overnight at 4°C. After washing immunoprecipitates 3 times in lysis buffer, RNA was extracted from the beads (Fisher Scientific cat. #A36797) and reverse transcribed to cDNA A (Qiagen cat. #74134). Ribosome pull-down was used to quantify the ribosome (18S)-to-*Ins1+2* mRNA ratio by qRT-PCR at each time point. The relative mRNA levels were determined by qRT-PCR with F: GCAATTATTCCCCATGAACG; R: GGGACTTAATCAACGCAAGC primers that recognize 18S; and F: CCTACCCCTGCTGGCCCTGCT; R: GTAGTTCTCCAGCTGGTAGAGGG primers that recognize both *Ins1* + *Ins2* mRNAs. The data were normalized to the 0 hour time point (n = 7 experiments but not all time points were used in all experiments).

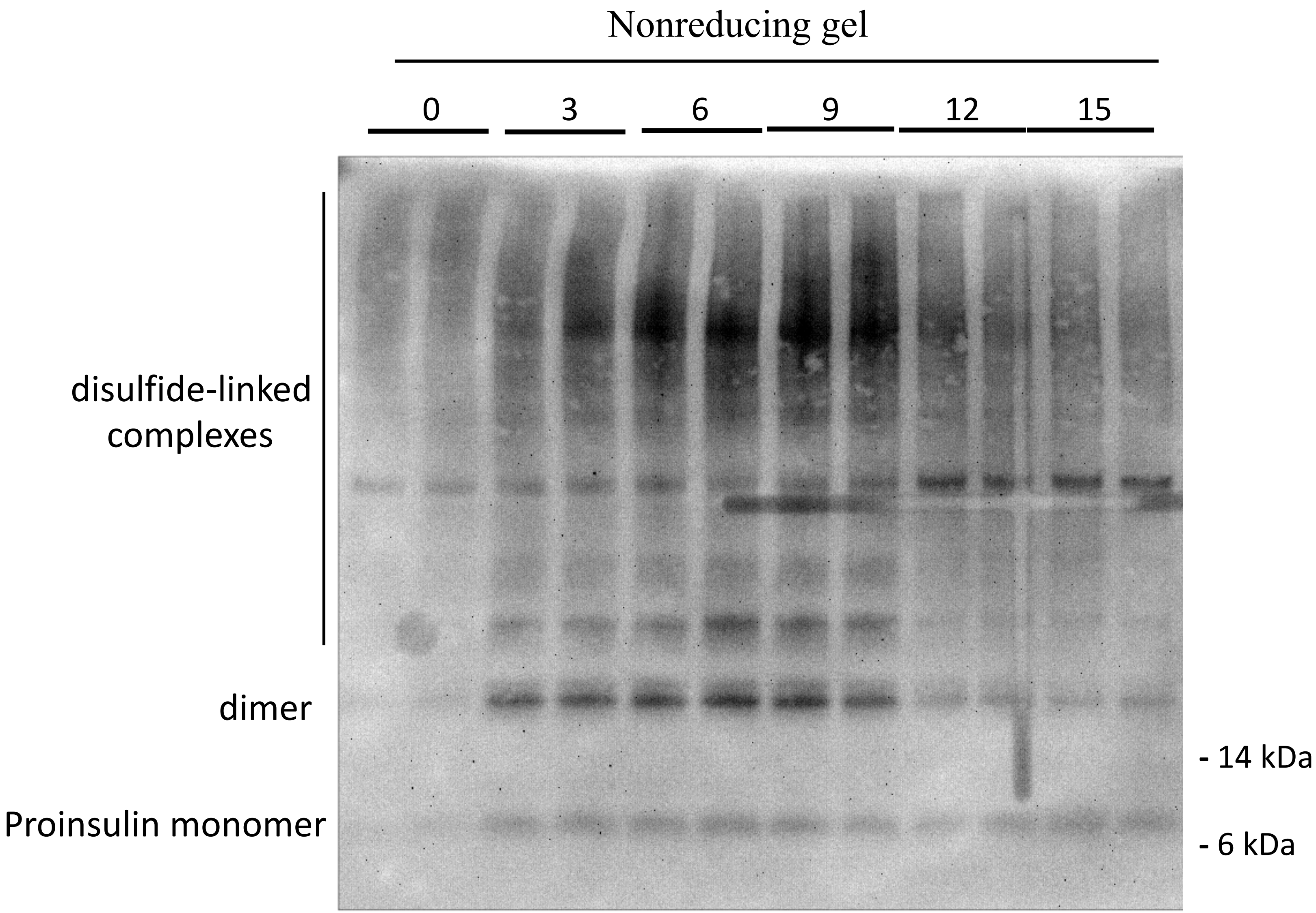

Xu et al., Supplemental Fig. S1, coupled to main Fig. 3A

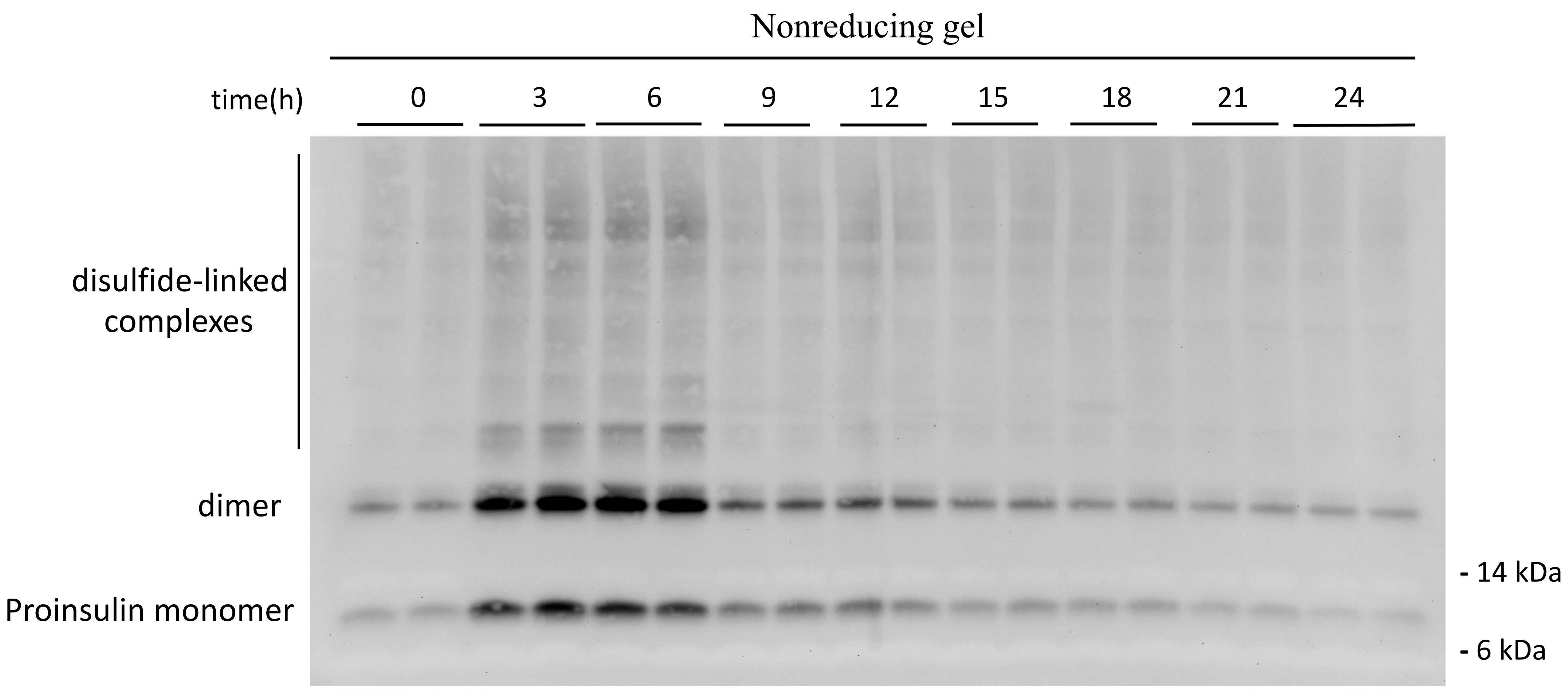

Xu et al., Supplemental Fig. S2, coupled to main Fig. 5A

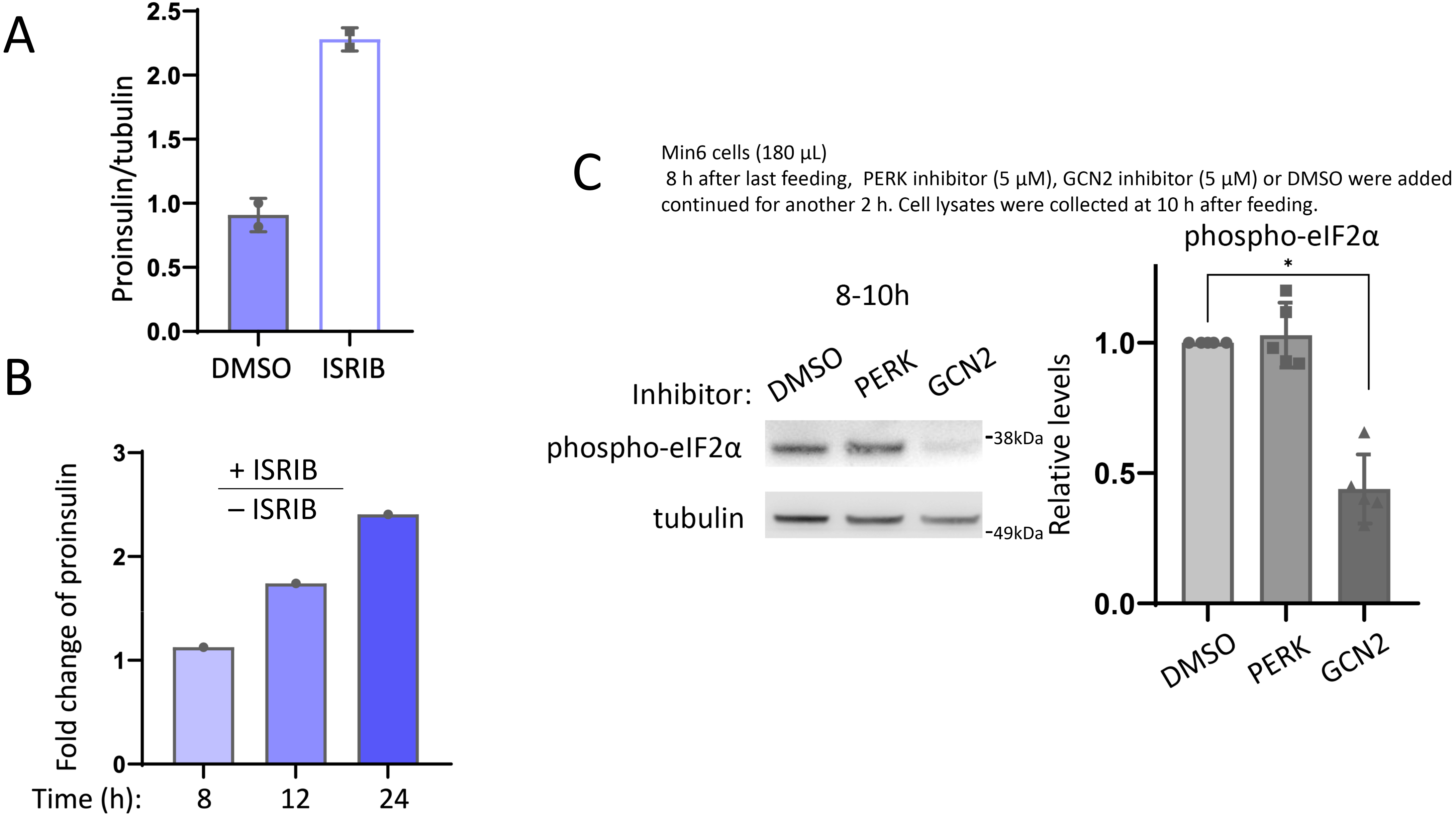

Xu et al., Supplemental Fig. S3, related to main Fig. 6

Min6 'Ribo-Tag' cells (RPL22-Flag)

18S mRNA levels normalized to ins1/2 mRNA

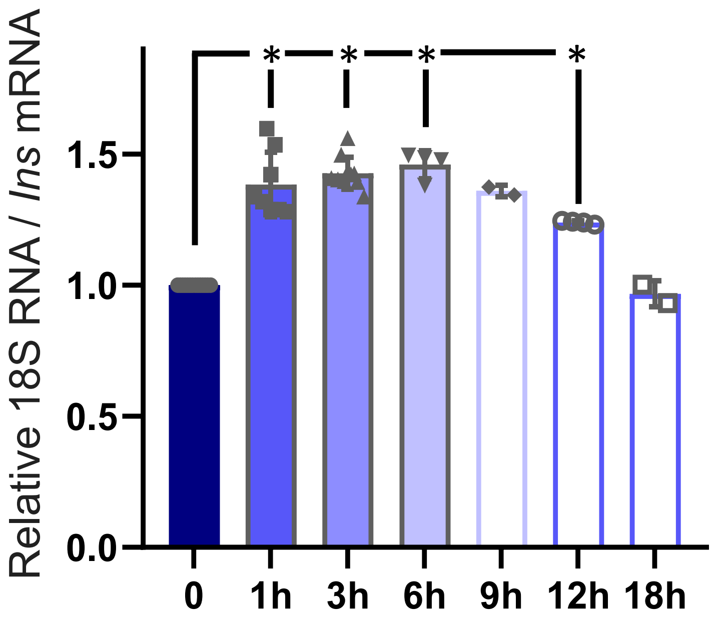

Xu et al., Supplemental Fig. S4, coupled to main Fig. 8
